# Advances in *Toxoplasma gondii* Vaccines: Current Strategies and Challenges for Vaccine Development

**DOI:** 10.3390/vaccines9050413

**Published:** 2021-04-21

**Authors:** Ki-Back Chu, Fu-Shi Quan

**Affiliations:** 1 Department of Biomedical Science, Graduate School, Kyung Hee University, Seoul 02447, Korea; kbchu@khu.ac.kr; 2 Medical Research Center for Bioreaction to Reactive Oxygen Species and Biomedical Science Institute, School of Medicine, Graduate School, Kyung Hee University, Seoul 02447, Korea; 3 Department of Medical Zoology, School of Medicine, Kyung Hee University, Seoul 02447, Korea

**Keywords:** *Toxoplasma gondii*, vaccine, immune response, protection

## Abstract

Toxoplasmosis, caused by the apicomplexan parasite *Toxoplasma gondii*, is one of the most damaging parasite-borne zoonotic diseases of global importance. While approximately one-third of the entire world’s population is estimated to be infected with *T. gondii*, an effective vaccine for human use remains unavailable. Global efforts in pursuit of developing a *T. gondii* vaccine have been ongoing for decades, and novel innovative approaches have been introduced to aid this process. A wide array of vaccination strategies have been conducted to date including, but not limited to, nucleic acids, protein subunits, attenuated vaccines, and nanoparticles, which have been assessed in rodents with promising results. Yet, translation of these in vivo results into clinical studies remains a major obstacle that needs to be overcome. In this review, we will aim to summarize the current advances in *T. gondii* vaccine strategies and address the challenges hindering vaccine development.

## 1. Introduction

*Toxoplasma gondii* has become a pathogen of global importance with 2 billion individuals being estimated to be infected with this Apicomplexan parasite [1]. In humans, the transmission of this parasite through the consumption of undercooked or contaminated food products can have severe consequences depending on the host’s immune system status. In immunocompetent healthy individuals, *T. gondii* infection tends to result in mild unspecific symptoms or asymptomatic as a whole, whereas their infection in immunocompromised or pregnant individuals incurs congenital toxoplasmosis which leads to premature abortion and ocular toxoplasmosis [2]. Currently, the gold standard therapeutic option for *T. gondii* infection involves the use of pyrimethamine and sulfadiazine [3]. However, there is a worrying trend of increased drug resistance in *T. gondii* which may exacerbate the disease severity and result in treatment failure [2]. Moreover, while drug treatment has been proven to be effective against *T. gondii* tachyzoites, they were virtually ineffective against tissue-encysted bradyzoites and latent stages of *T. gondii* [4]. In 2015, the financial scandal involving pyrimethamine caused a massive uproar. With the cost of pyrimethamine undergoing a 5000% increase overnight from $13.50 to $750 per pill, alternative options to deal with toxoplasmosis have become a necessity since this generic drug has become more or less unaffordable [5]. Vaccination is widely considered as the most cost-effective method of disease prevention and has a tremendous influence on a socioeconomic level [6]. Given these circumstances, an efficacious vaccine that prevents toxoplasmosis would be a major medical advancement and greatly benefit the globe.

Toxovax, a live-attenuated vaccine based on the tachyzoites of *T. gondii* S48 strain, is currently the only commercially available toxoplasmosis vaccine [7]. Unfortunately, there are several limitations to this vaccine. First, the vaccine usage and administration are strictly limited to veterinary purposes and have an extremely short shelf life of 10 days [7]. Secondly, because the vaccine is based on a live-attenuated pathogen, its administration in humans is impossible due to ethical reasons and safety concerns. In particular, attenuated vaccines are capable of reverting to its virulent wild-type form and cause diseases that it was meant to prevent, hence completely defeating the purpose of the vaccine. As such, developing an effective toxoplasmosis vaccine suitable for humans is highly desired. In this review, we will discuss the recent progress in the development of toxoplasmosis vaccines using DNA, protein, nanoparticles, live-attenuated, and carbohydrate vaccine platforms and address their accomplishments in animal models as well as some of their outlying concerns.

## 2. Vaccine Platforms against *T. gondii*

### 2.1. DNA and Vectored Vaccines

DNA vaccines are perhaps one of the most efficient vaccine platforms reported to date. Some of the key features that continue to fuel their research stems from their low production cost, ease of production, capability for inducing both humoral and cellular immune response, etc. [8]. To date, multitudes of *T. gondii* DNA vaccine studies have been conducted. Nevertheless, the results have been more or less conflicting. Specifically, while majority of the vaccines triggered antibody responses and production of the cytokine IFN-γ, the degree to which vaccines protected mice varied as indicated by survival and brain cyst counts. The vast majority of the *T. gondii* DNA vaccine studies were conducted using several well-characterized *T. gondii* virulence factors, such as the rhoptry proteins (ROP), dense granule proteins (GRA), microneme proteins (MIC), and the surface antigens (SAG). Immunizing the mice with vaccines expressing ROP1 induced a potent Th1 immune response that provided partial protection upon a lethal challenge with *T. gondii* RH strain [9]. ROP13 DNA vaccine immunization enhanced IL-17 and IL-22 mRNA expressions and limited the circulating parasite load following an RH challenge in the blood of mice [10]. Interestingly, the level of parasite inhibition demonstrated by this vaccine was similar to those induced by *T. gondii* lysate antigen (TLA) immunization [10]. Immunization with *T. gondii* ROP16 DNA vaccine expressed using the canine adenovirus significantly bolstered the production of cytokines IFN-γ, IL2-, and IL-4, and partially protected mice against RH challenge infection [11]. Similarly, subcutaneous and intranasal ROP18 vaccine administration induced a mixed Th1/Th2 systemic immune response as indicated by the production of IFN-γ, IL2-, and IL-5 cytokines in mice [12]. Nevertheless, brain cyst reduction was only observed when this vaccine was administered through the intranasal route upon a challenge infection with the Type II 76K strain, and neither vaccination route induced significant enhancements to CD8^+^ T cell proliferation [12]. DNA vaccine encoding the ROP54 reduced the brain cysts by 35.9% and also significantly prolonged the survival of immunized mice following a PRU and RH strain challenge, respectively [13]. Immune protection induced by the ROP21 vaccine was similar to previous studies, characterized by high antibody titers and IFN-γ levels and partial protection against both RH and PRU strains [14]. 

**Figure 1:**
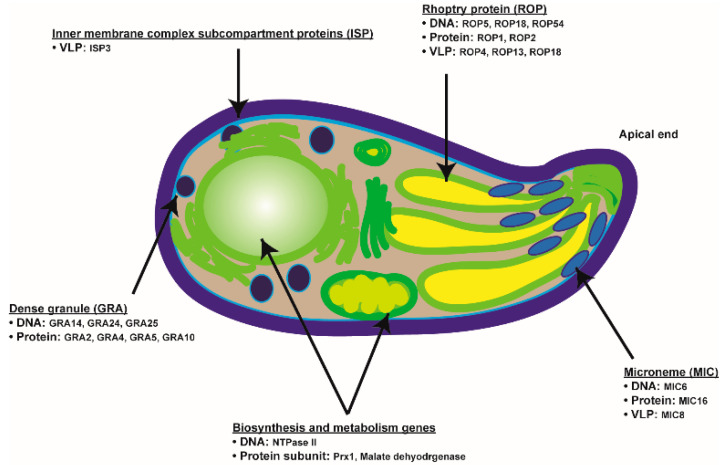
Brief diagram depicting *T. gondii* target antigens and the vaccine platforms used to assess their efficacies in animal models.

Studies using GRA2 and GRA5 DNA vaccines also revealed that both of these vaccines elicited potent Th1 cytokine responses while marginally prolonging survival post-challenge with the RH strain [15]. While significantly enhanced IL-4 and IL-10 cytokine productions were observed, their quantities were considered relatively low in comparison to IL-2 and IFN-γ levels [15]. GRA16 DNA vaccine induced a drastic increase in IL-2, IFN-γ, and IL-10 cytokine levels while a marginal increase in IL-4 was observed. Although their enhancements did not contribute to prolonging the survival of immunized mice, noticeable reductions in brain cyst counts were observed [16]. GRA24 DNA vaccine immunization, characterized by a marked increase in cytokine productions, T cell proliferation, and antibody responses drastically prolonged the survival of immunized mice following a challenge infection with a lethal dose of RH strain [17]. Several well-characterized genes have been widely incorporated as vaccine components in multiple vaccine platforms as illustrated in Figure 1.

Apart from these well-characterized antigens, several other antigens were investigated as a potential vaccine candidate antigen. A vaccination study using the plasmid DNA vaccine expressing the heat shock protein (Hsp) 40 reported that its immunization partially reduced the brain cyst burden and prolonged the survival of mice challenged with PRU and RH strains, respectively, despite failing to induce parasite-specific antibody responses and Th1/Th2-associated cytokines [18]. DNA vaccine encoding the Myc regulation 1 (MYR1) induced both humoral and cellular immune responses contributing to prolonged survival of immunized mice post-challenge with RH strain [19]. Similar to vaccines designed using the ROP or the GRA family antigens, mice immunized with the MYR1 DNA vaccine demonstrated elevated antibody responses, CD4^+^ and CD8^+^ T cells, and several cytokines associated with Th1 and Th2 immunity [19]. Vaccines constructed using the *T. gondii* calcium-dependent protein kinases (CDPK) partially protected mice against an RH strain challenge infection. Both of the vaccines constructed using CDPK1 [20] and CDPK2 [21] conferred a similar degree of protection, as indicated by prolonged survival and high levels of IFN-γ, IL-12(p70), and IL-10 production. *Doc2*, a gene involved in calcium signaling, has been investigated as a vaccine target. DNA vaccine expressing this gene significantly prolonged the survival in Kunming mice following an RH challenge infection and drastically lessened the brain cyst burden post-challenge with PRU strain [22].

Adjuvanting the DNA vaccines was found to enhance the vaccine-induced protective immune responses. The GRA14 DNA vaccine, although it successfully induced antibody responses and enabled lymphocyte proliferation as well as inducing Th1 cytokines, merely prolonged the survival of mice by a few days following a lethal challenge with RH strain [23]. Supplementing this GRA14 DNA vaccine with a calcium phosphate nanoparticle adjuvant increased the *T. gondii*-specific IgG1 and IgG2a antibody responses as well as lymphocyte proliferation, albeit failing to result in enhanced survival [24]. Vaccines expressing the B and T cell epitopes of ROP29 adjuvanted with resiquimod (R848), an agonist of Toll-like receptor (TLR) 7/8, enhanced the antibody responses and Th1 cytokine production as well as further reducing the brain cyst burden compared to the unadjuvanted control group post-challenge with PRU strain [25]. A multi-epitope ROP8 vaccine co-delivered with the genetic adjuvant IL-12 promoted antibody production and Th1 immune responses, with a marginal increase in survival [26]. Compared to single antigen vaccines or unadjuvanted multi-antigen DNA vaccines, adjuvanted multi-antigen vaccines encompassing SAG1 and GRA7 conferred the highest degree of protection against a lethal RH challenge [27]. An ROP5 and ROP18 DNA cocktail vaccine combined with the molecular adjuvant IL-33 significantly enhanced the protective efficacy of the vaccines against challenge infections with RH and PRU strains, indicated by the prolonged survival time of 30 days and minimal brain cysts, respectively [28]. A DNA vaccine encompassing SAG1 and ROP2 adjuvanted with the hepatitis B virus (HBV) antigen evoked an immune response contributing to the survival of immunized mice, while the unadjuvanted control groups perished by 9 days post-infection (dpi) [29]. Interestingly, unadjuvanted vaccines expressing ROP13 and GRA14 conferred better protection against *T. gondii* infection in the absence of adjuvants [30]. While alum-adjuvanted ROP13+GRA14 vaccines contributed to higher Th2 response induction in mice, a marginally extended survival rate was observed from mice immunized with unadjuvanted ROP13+GRA14 vaccines [30].

DNA vaccines expressing multiple antigens generally conferred better protection against *T. gondii* than single antigen vaccines (Table 1). A cocktail DNA vaccine comprising *T. gondii* profilin, ROP16, ROP18, microneme protein (MIC) 6, and CDPK3 conferred substantial protection against the PRU strain, as indicated by heightened Th1-associated cytokines and diminished brain cysts [31]. Notably, marginally higher brain cyst reduction was detected from mice immunized with the vaccine expressing all five aforementioned antigens than those expressing only four out of the five antigens [31]. Consistent with these findings, a multi-antigenic DNA vaccine expressing GRA24, GRA25, and MIC6 induced the highest level of protection compared to the single antigen or GRA24 and GRA25 combined vaccines [32]. A single dose of hexaplex modified dendrimer nanoparticle vaccine carrying replicons encoding GRA6, ROP2A, ROP18, SAG1, SAG2A, and AMA1 successfully protected mice against a lethal challenge infection with PRUΔ*hxgprt* strain [33]. Multi-epitope DNA vaccine formulated by fusing the surface antigen 1 (SAG1) with ROP2 and delivered using the hepatitis B virus core antigen significantly enhanced antibody production and splenic IFN-γ production in mice [34]. Striking differences in protection against low and high RH infective doses were observed from mice immunized with recombinant adenovirus vaccine expressing MIC3, ROP9, and SAG2 antigens [35]. Contrary to the findings above, the number of antigens displayed by a vaccine did not necessarily correlate with the highest level of protection. Evidently, the highest survival rate was observed from mice immunized with ROP9 and MIC3-expressing vaccines, which even surpassed the survival rate demonstrated by the SAG2-ROP9-MIC3 immunization group [35]. Regardless of these discrepancies, the consensus appears to be that superior protective efficacy can be observed from multi-antigenic vaccines.

The method of vaccine delivery and vaccination routes also impacted the efficacy of DNA vaccines. Immunizing mice with a suicidal nucleic acid vaccine expressing the *T. gondii* nucleoside triphosphate hydrolase II (NTPase II) resulted in a Th1 predominant immune response which partially protected against RH Δ*Ku80* and PRU strains [36]. When encapsulated in lipid nanoparticles, NTPase II vaccine efficacy was greatly enhanced against both RH and PRU strains. Notably, 20% of the mice immunized with nanoparticle-encapsulated vaccines survived post-challenge with RH strain whereas all of the mice immunized with a non-encapsulated NTPase II vaccine perished 15 dpi [37]. Similarly, mice immunized with the *T. gondii* profilin-expressing DNA vaccine delivered using lipid nanoparticles were protected from a challenge infection with PLK strain [38]. Different routes of vaccine administration induced different levels of protection. Upon immunizing mice with recombinant adenovirus vaccines expressing ubiquitin-conjugated multi-stage antigen segments, it was demonstrated that mucosal immunization routes were far more effective at conferring protection than systemic immunization routes [39]. 

Vaccination regimen also had a profound effect on regulating parasite burden in mice. Different immunization regimens have been an effective method of inducing protection in animal models. Priming mice with a multi-epitope vaccine adjuvanted with glucopyranosyl lipid adjuvant-stable emulsion (GLA-SE), followed with multi-epitope polypeptide protein vaccine boost enhanced IFN-γ-producing memory CD8^+^ T cell population and limited the cyst load following an ME49 strain challenge infection [40]. The protective efficacy of a *T. gondii* DNA vaccine expressing the secreted protein with an altered thrombospondin repeat (SPATR) induced a mixed Th1/Th2 response and significantly prolonged the survival of mice post-challenge with *T. gondii* RH strain [41]. Mice immunized with the SAG4 DNA vaccine along with its B/T cell epitope polypeptides elicited antibody responses and Th1-dominant immune responses, in addition to prolonging survival and reducing brain cysts against lethal RH and PRU strain challenges, respectively [42]. GRA14 immunization via DNA prime and protein boost coated with calcium phosphate further prolonged the survival of mice infected with RH strain than prime-boost immunization with DNA vaccine only [43].

### 2.2. Protein and Recombinant Subunit Vaccines

Protein vaccines incorporate a highly purified antigen as the vaccine component. Due to this, protein and subunit vaccines are extremely safe and chances of side effects occurring in recipients are low. However, as with DNA vaccines, their immunogenicity pales in comparison to live-attenuated vaccines and for this platform to be used, a specific antigen involved in disease pathogenesis must be identified [44]. Similar to the *T. gondii* DNA vaccines, a wide array of antigens have been explored for potential vaccine candidacy ranging from the aforementioned well-characterized *T. gondii* genes to crude extract lysate antigens, as well as other proteins involved in biosynthetic pathways (Table 2). Irradiated *T. gondii* tachyzoite antigen extracts were found to be capable of priming the immune system, with potent humoral and cellular immunity being induced to confer protection against RH and ME49 strains in mice [45]. Irradiating the *T. gondii* soluble antigens with a high radiation dose conferred a similar level of protection to those induced by irradiated RH tachyzoites [46]. While the effects of radiation from both were more or less similar, as indicated by identical levels of peripheral B cell inductions in blood and survival rates, an irradiated soluble antigen induced greater percentage of CD4^+^ T cells whereas irradiated RH tachyzoites had a greater influence on CD8^+^ T cell induction [46]. The efficacy of lysate antigen-based vaccines was further assessed in swine. Consistent with the findings from mice, vaccines designed using the *T. gondii* lysate antigens adjuvanted with QuilA reduced the parasite burden in the muscle tissues of swine [47].

As demonstrated when using DNA vaccines, antigens delivered using nanoparticles have been reported to be promising. When *T. gondii* extract antigens were delivered using porous nanoparticles, its immunization in mice elicited a potent Th1/Th17 cellular immunity and ensured that all of the immunized mice survived the lethal challenge with *T. gondii* 76 K strain with drastically reduced brain cyst counts [57]. Nanospheres loaded with *T. gondii* histone H2A1 protected mice against the virulent RH strain by prolonging survival and enhanced the production of Th1 cytokines in a dose-dependent manner [58]. When vaccines with multiple antigen components were delivered using nanoparticles, significant enhancements to vaccine efficacies were observed. A single dose of chimeric protein vaccine comprising SAG1 and SAG2 encapsulated in poly(lactide-co-glycolide (PLGA) conferred protection against a lethal challenge infection with RH tachyzoites in mice [59]. Similarly, the *T. gondii* vaccine expressing the T and B cell epitopes of AMA1, GRA4, ROP2, and SAG1 delivered in PLGA nanoparticles invoked potent Th1 responses in mice and conferred better protection than the identical vaccine adjuvanted with alum [48]. GRA10 epitope delivery in chitosan-based nanoparticles induced Th1-dominant immune responses in mice and conferred partial protection against both acute and chronic toxoplasmosis [49].

Interesting results have been reported from subunit vaccines expressing stress response-associated proteins. A peroxiredoxin 1-based *T. gondii* vaccine triggered the production of IL-12p40 and IL-6 cytokines in murine peritoneal macrophages and promoted survival of mice post-challenge with the PLK strain while severely restricting parasite replication [50]. Immunizing mice with a *T. gondii* Hsp70 subunit vaccine combined with Alum neither altered the cytokine profiles nor exerted any functional effect on the parasite by itself. Yet, the vaccine still managed to confer partial protection in mice challenged with the ME49 strain via oxidative stress regulation involving iNOS expression [60]. *T. gondii* vaccines expressing the macrophage migration inhibitory factor (MIF) significantly protected immunized mice against acute and chronic toxoplasmosis upon a challenge infection with RH tachyzoites and PRU cysts [51]. 

Biosynthetic enzymes and other proteins involved in several signaling pathways have also been used as target antigens for the *T. gondii* vaccine, but their results varied widely. Partial protection against RH strains was observed from mice immunized with the parasite’s malate dehydrogenase [52] or *T. gondii* actin depolymerizing factor [53], as both of these studies ensured the survival of approximately 40% of the immunized along with significant parasite burden reductions in the liver and the brain. The recombinant *T. gondii* CDPK3 vaccine elicited partial protection against RH tachyzoite and a PRU challenge infection in mice, characterized by high antibody titers and a Th1-dominant immune response [54]. Protective efficacy of tyrosine hydroxylase-based vaccines has been explored, but the protection induced was mild in spite of the humoral and cellular immunity induced through immunization [55]. In one study, *T. gondii* vaccines expressing extracellular signal-related kinases were used to immunize the mice on a weekly basis for 5 consecutive weeks. This Freund’s adjuvant-supplemented vaccine imparted partial protection against both GT1 and PRU challenge infections [56].

Protective efficacy of multi-antigenic subunit vaccines has also been investigated. Subunit vaccines comprising of GRA2 and GRA5 strongly enhanced Th1-associated cytokine response and prolonged survival against an RH tachyzoite challenge infection in mice [61]. Synthetic B and T cell epitopes of GRA2 can partially protect immunized mice against an ME49 challenge infection [62]. Chimeric protein vaccine comprising fragments of AMA1 fused to SAG1, GRA2, and ROP1 were assessed, with all of the generated chimeric vaccines conferring decent levels of humoral and cellular immune responses [63]. Similarly, protein vaccines based on B and Th cell epitopes of *T. gondii* aspartic protease 3 promoted the production of Th1 cytokines and prolonged survival for as long as 18 days after a challenge infection with the virulent RH strain [64]. MIC16 expressed on the surface of the yeast *Saccharomyces cerovisiae*, elicited both humoral and cellular immune responses after 3 immunizations which protected mice against a lethal dose of RH tachyzoites [65]. SAG1 peptide adjuvanted with the plant *Nicotiana benthamiana* Hsp90 significantly reduced the brain cyst load and conferred partial protection in immunized mice challenged with ME49 strain [66]. 

Dendritic cells are of keen interest for improving vaccine efficacy as their targeting has been demonstrated to be an effective method against chronic toxoplasmosis. SAG1 targeting of DEC205 endocytic receptor on dendritic cells via single-chain fragment variable antibody enhanced both cellular and humoral immune responses in mice, which contributed to strongly inhibiting brain cyst formation [67]. Effective bone marrow dendritic cell activation was observed from multi-antigen protein vaccines comprising GRA7 and *T. gondii* profilin compared to either antigen alone, and this enabled effective induction of humoral and cellular immunity [68].

### 2.3. Nanoparticles, Virus-Like Particle Vaccines

Self-assembling nanoparticle vaccines have recently emerged as a novel vaccine platform for *T. gondii* vaccine design. Generally, nanoparticles have been widely utilized as vaccine carriers. By incorporating this strategy, antigens that would otherwise undergo proteolytic degradation are protected, thus ensuring their successful uptake by antigen-presenting cells for immune response induction [69]. At present, only a few studies have explored their potential as *T. gondii* vaccines. Though limited in number, the results seem to point towards the fact that these vaccines are highly efficacious. A multi-epitope nanoparticle vaccine adjuvanted with the GLA-SE stimulated IFN-γ secretion and conferred robust protection against a Type II *T. gondii* ME49 challenge infection in HLA transgenic mice [70]. Similarly, a chimeric polypeptide vaccine expressing the CD4 T cell, CD8 T cell, and B cell epitopes emulsified in GLA-SE conferred protection against several different HLA transgenic mice [71]. Intranasal administration of maltodextrin-based nanoparticle vaccines formulated with total *T. gondii* antigen extracts was highly protective against chronic and congenital toxoplasmosis in mice [72]. The same vaccine was later assessed in sheep and was also confirmed to be effective at preventing latent and congenital toxoplasmosis in ewes [73]. 

Previous studies have investigated the anti-parasitic effect of metallic nanoparticles. Nanoparticles based on inorganic metals such as gold, silver, and platinum were reported to demonstrate an anti-*T. gondii* effect whose mechanism of action appeared to involve altering the redox potential of the parasite [74] and several metallic alloys were also confirmed to restrict the growth of *T. gondii* in vitro [75]. Administering biogenic silver nanoparticles reduced the proliferation of *T. gondii* by recruiting inflammatory mediators in trophoblast cell lines, whereas the parasitic reductions occurred independently of inflammatory mediators in chorionic villi [76].

Virus-like particles (VLPs) are another novel approach to vaccine development. VLP-based vaccines have underwent clinical trials for several viral diseases with fascinating results and are commercially available for clinical use [77]. VLP vaccines are of keen interest and their further development has been warranted for good reasons. As VLPs are completely devoid of genetic material required for replication, they are extremely safe. Additionally, due to their size, enabling rapid VLP trafficking into the lymph nodes a rapid immune response induction is feasible. Moreover, the repetitive antigen presentation on the particle surface promotes potent immune response induction [78]. As with nanoparticle vaccines, *T. gondii* vaccine studies conducted using VLPs are limited. Some of the earliest VLP constructs displayed the inner membrane complex subcompartment protein 3 (ISP3) of *T. gondii*, which conferred protection in mice challenge-infected with the *T. gondii* ME49 strain through the intraperitoneal [79] or oral route [80]. Following suit, multiple studies have been conducted investigating the efficacy of VLP vaccines against ME49 strains using several well-characterized *T. gondii* antigens (Table 3). VLPs expressing either ROP4 or ROP13 *T. gondii* completely protected mice against a lethal dose of ME49 cysts and alleviated the inflammatory responses in their brains [81]. The protective efficacy of these VLPs was further enhanced when the two antigens were co-expressed on a single VLP, as it conferred 100% survival against a lethal challenge with ME49 strain and drastically reduced the cyst counts in mice [82]. Similar findings were reported from mice immunized with multi-antigenic VLPs expressing IMC, ROP18, and MIC8 [83]. VLPs solely expressing the AMA1 protein were not as effective as the VLPs expressing the rhoptry proteins as the protective efficacies were moderate at best [84]. 

Supplementing VLP vaccines with adjuvants, as with other vaccine platforms, strengthened the protective efficacy of the vaccines. Multi-antigenic VLPs adjuvanted with the Toll-like receptor 9 agonist CpG ODN enhanced both humoral and cellular immune responses in mice and further limited cyst formation in comparison to unadjuvanted VLPs [85]. Using the identical VLP vaccine, increasing the number of immunizations was also effective at reducing the cyst counts [86].

While a large majority of the VLP-based vaccines were targeted against the ME49 strain of *T. gondii*, only a few studies have been conducted against type I *T. gondii* strains. Surprisingly, intranasal administration of a VLP vaccine expressing the MIC8 of *T. gondii* conferred 100% survival when challenged with the RH strain, while intramuscular administration of the identical VLP vaccine conferred 60% survival in mice [89]. Co-immunizing VLPs with ROP18 and MIC8 significantly enhanced survival in mice when challenge-infected with the GT1 strain [87]. A multi-antigen VLP vaccine expressing IMC, ROP18, and MIC8 almost completely inhibited parasite replication and prolonged survival of mice following a GT1 challenge infection in mice [90]. Chimeric VLP constructed using the T cell and B cell epitopes on the hepatitis B virus core antigen conferred partial protection against RH and PRU strains [88]. 

### 2.4. Live-Attenuated Vaccines

Safety concerns regarding live-attenuated vaccines continue to exist and serve as a barrier for clinical trials, but their efficacies remain phenomenal. Notably, compared to other vaccine platforms, live-attenuated *T. gondii* vaccines conferred near-complete protection against multiple *T. gondii* strains as described in Table 4. A vast majority of the experimental live-attenuated vaccines were designed targeting the biosynthetic pathways of *T. gondii*. Deleting carbamoyl phosphate synthetase II, an enzyme crucial for pyrimidine biosynthesis in *T. gondii* RH strain, conferred partial protection in mice [91]. Immunizing mice with the α-amylase knockout type II ME49 mutant invoked both cellular and humoral immune response and inhibited parasite proliferation [92]. Mice immunized with the adenylosuccinate lyase-deficient ME49 were protected upon a challenge infection with the type I RH, type II ME49, and type III VEG strains [93]. Genetically engineering *T. gondii* using the CRISPR-Cas9 system to construct a *T. gondii* RH strain with a defective novel putative transporter 1 conferred heterologous protection and ensured 100% survival following a challenge infection with RH, PYS, and PRU strains [94]. *T. gondii* RH strain with defective *Gra17* gene mutant as an attenuated vaccine provided partial protection against homologous and heterologous *T. gondii* strains [95].

Mice immunized with *T. gondii* RH tachyzoites with deleted apical membrane antigen 1 (AMA1) conferred long-lasting protective immunity against both homologous and heterologous challenge infections [96]. Immunizing mice with a *T. gondii* PRU strain with disrupted orotidine 5′-monophosphate decarboxylase (*Ompdc*) gene also conferred homologous and heterologous protection against both acute and chronic toxoplasmosis [97]. An attenuated ME49 strain lacking both of the lactate dehydrogenases 1 and 2 genes efficiently protected mice against a wide variety of type I, II, and III *T. gondii* strains [98].

Earlier studies have reported the potential of attenuated vaccines for preventing acute, chronic, and congenital toxoplasmosis. An attenuated PRU strain with deficient *cdpk2* gene was reported to protect mice from acute, chronic, and congenital toxoplasmosis following heterologous challenge infections with RH, PYS, and TgC7 strains [99]. A similar degree of protection was observed from mice immunized with *T. gondii* RH strain lacking tyrosine kinase-like 1 (*Tkl1*) [100] and also from RH tachyzoites lacking GRA17 and novel putative transporter (*Npt1*) genes [101]. 

Live-attenuated vaccines were generally highly protective when tested in murine models, and this phenomenon was also observed in other animals. Felines immunized with microneme (MIC) 1 and 3 double knock out *T. gondii* mutant induced *T. gondii*-specific antibody response but failed to prevent oocyst shedding [102]. A *T. gondii* mutant lacking the fertilization factor HAP2 was demonstrated to be a potential live-attenuated vaccine candidate. Inoculating this mutant parasite into cats completely prevented oocyst excretion upon a subsequent challenge with wild-type *T. gondii* and inhibited the establishment of systemic infection [103].

### 2.5. Carbohydrate Vaccines

Vaccines designed using the glycosylphosphatidylinositol (GPI) glycoconjugates were considered to be potential vaccine candidates, as they confer favorable advantages over the traditional vaccine systems. Notably, compared to the live-attenuated or the inactivated vaccines, they are much safer to use. Moreover, because mutations in these essential biosynthetic genes have devastating consequences for the parasite, resistance against these carbohydrate antigens is not likely to occur [104]. To date, *T. gondii* vaccine research conducted using carbohydrate or glycolipid-based vaccines is extremely rare. Recently, a study by Götze et al. [105] investigated the protective efficacy of GPI-based vaccines against *T. gondii* infection in mice. Although robust antibody responses were induced in mice post-immunization, they were not specific to the carbohydrate antigen and failed to confer adequate protection upon a lethal challenge with *T. gondii* tachyzoites [105]. Vaccines based on naturally occurring polysaccharides such as chitosan have been investigated. In one study by Teimouri et al. [106], it was reported that chitosan nanoparticles of various molecular weights were capable of inhibiting *T. gondii* RH tachyzoites. Further studies investigating methods that enhance the immunogenicity and protective efficacy of these carbohydrate vaccines could enable their clinical application. 

## 3. Limitations and Challenges to Vaccine Design

Vaccines against toxoplasmosis can be challenging to develop as numerous factors must be taken into consideration for each of the vaccine platforms. There are lingering safety concerns involving the use of DNA vaccines, such as the potential genomic integration of the plasmid which may activate oncogenic proteins and antibody generation against the vaccine DNA itself [107]. While these safety issues are of concern, they are not the major factors hindering its emergence as commercially viable prophylaxis tools since the probability of these unintended side effects occurring in vaccine recipients is low [108]. Rather, the Achilles heel of DNA vaccines has been its low immunogenicity [109]. To this end, applying several strategies such as adjuvant inclusion could result in their clinical approval in the foreseeable future. Recombinant subunit vaccines and virus-like particles are safe but as with DNA vaccines, they are less immunogenic especially when compared to live-attenuated vaccines. Additionally, in the latter of the two, a high production cost and additional down-stream purification processes that delay production speed are some of the disadvantages associated with this method [110]. Carbohydrate-based vaccines, as with the aforementioned vaccine platforms, also faces issues involving poor immunogenicity and antibody affinity. As such, careful selection of carrier protein and conjugation methods are necessary to enhance its immunogenicity [111].

While live-attenuated *T. gondii* vaccines are successful and proven to be highly immunogenic in several animals, these cannot be tested in humans due to ethical issues as the possibility of attenuated vaccines reverting to their virulent wild-type form cannot be neglected. For example, safety concerns involving the use of live-attenuated oral poliovirus vaccine have been on the rise over the past few decades. The reversion of these attenuated vaccines can be fatal, especially in areas with low vaccination coverage, as it contributes to vaccine-derived poliovirus outbreaks resulting in severe cases of paralytic poliomyelitis [112]. This is particularly true for attenuated *T. gondii* RH or PRU strains with a deficient *Ku80* gene, whose involvement in DNA repair renders the parasite more susceptible to accumulating genetic mutations over time [113]. Moreover, in real world field settings, individuals can be exposed to multiple strains rather than a pre-defined parasite strain which renders its efficacy somewhat questionable [114]. While the three clonal lineages described earlier by Howe and Sibley [115] provided a general understanding of the population genetic structure of *T. gondii* and its correlation to clinical toxoplasmosis, this was considered to be not a true global representation of the parasite as the clinical samples reported were predominantly from the western hemisphere [116]. Evidently, a highly virulent atypical strain was isolated from a clinical case of congenital toxoplasmosis in Europe, which signified that the immunity against the clonal lineages prevalent in Europe may not confer protection against these atypical strains [117]. Currently, numerous atypical strains have been reported throughout the globe and several of these are estimated to possess greater pathogenicity than those reported from the clonal types [116]. Based on this notion, vaccines conferring cross-strain protective immunity should also be investigated.

## 4. Conclusions and Future Perspective

Progress in toxoplasmosis vaccine development has been ongoing for decades, but an effective vaccine for clinical use is still lacking. While various strategies have been utilized for vaccine development, variances in vaccine efficacies exist across multiple vaccine platforms. The consensus seems to be that multi-antigenic vaccines tend to be more efficacious than single antigen-expressing vaccines, irrespective of the vaccine platform used. Based on this rationale, careful selection of highly immunogenic antigens and combining them to construct a multi-antigenic vaccine on platforms such as VLPs would advance the current progress in *T. gondii* vaccine design and benefit both humans and other intermediate hosts. Alternatively, the possibility of oral vaccines for immunizing non-human intermediate hosts could be explored. Since oral vaccines are easy to administer and enable mass vaccinating to be feasible, developing an ingestible vaccine using *T. gondii* antigen-expressing recombinant baculoviruses is plausible. In summary, with continued multi-disciplinary efforts and the application of diverse strategies, developing a successful toxoplasmosis vaccine may be feasible in due time.

## Figures and Tables

**Table 1:** An overview of the *T. gondii* DNA vaccines and their efficacies. Only the studies that assessed all four of the parameters listed below were selected and referenced in the table. Survival refers to the total duration of survival for immunized mice. +, weak; ++ intermediate; +++ strong.

Vaccine Platform	Animal Model	Antibody Response	Cellular Immune Response	Parasite Burden Reduction	Survival	Reference
DNA vaccine	Mouse					
ROP54		+	++	+	RH: 15 days	[13]
GRA16		+	++	+	RH: 10 days	[16]
HSP40		+	+	+	RH: 10 days	[18]
DOC2C		+	++	+	RH: >15 days	[22]
GRA14		++	+	++	RH: >8 days	[23,24]
ROP5+ROP18		+++	++	++	RH: >28 days	[28]
GRA24+GRA25+MIC6		++	+++	++	RH: >20 days	[32]
NTPase II		+	++	+	RH: 18 days, 20%	[36,37]

**Table 2:** An overview of the *T. gondii* protein and subunit vaccines and their efficacies. Only the studies that assessed all four of the parameters listed below were selected and referenced in the table. Survival refers to the total duration of survival for immunized mice. +, weak; ++ intermediate; +++ strong.

Vaccine Platform	Animal Model	Antibody Response	Cellular Immune Response	Parasite Burden Reduction	Survival	Reference
Protein subunit						
Multiantigenic epitopes		++	+	+	RH: 16 days	[48]
Multi-epitope		++	+	++	RH: 21 days	[49]
Prx1		+++	+	+	PLK: >60%	[50]
MIF	Mouse	+++	+	++	RH: >30%	[51]
Malate dehydrogenase		++	++	++	RH: 50%	[52]
Actin depolF		++	+	+	RH: 40%	[53]
CDPK3		+++	+	+	RH: 16 days	[54]
Tyrosine hydroxylase		+++	++	+	RH: 18 days; PRU: 50%	[55]
ERK7		+	++	+	RH: 19 days; PRU: 50%	[56]

**Table 3:** An overview of the *T. gondii* virus-like particle vaccines and their efficacies. Only the studies that assessed all four of the parameters listed below were selected and referenced in the table. Survival refers to the total duration of survival for immunized mice. +, weak; ++ intermediate; +++ strong.

Vaccine Platform	Animal Model	Antibody Response	Cellular Immune Response	Parasite Burden Reduction	Survival	Reference
Virus-like particles						
ROP4, ROP13		++	++	+++	ME49: 100%	[82]
IMC, ROP18, MIC8	Mouse	++	+	+++	ME49: 100%	[85,86]
ROP18+MIC8		++	+	++	GT1: 17 days	[87]
Multi-antigenic epitope		+	+	+	RH: 20 days	[88]

**Table 4:** An overview of the live-attenuated *T. gondii* vaccine platforms and their efficacies. Only the studies that assessed all four of the parameters listed below were selected and referenced in the table. Survival refers to the total duration of survival for immunized mice. +, weak; ++ intermediate; +++ strong.

Vaccine Platform	Animal Model	Antibody Response	Cellular Immune Response	Parasite Burden Reduction	Survival	Reference
Live-attenuated						
Δα-amylase		++	++	++	RH, ME49, VEG, C7719,	[92]
					WH1: >90%	
ΔAdsl	Mouse	++	+++	++	RH, VEG, ME49: 100%	[93]
ΔNpt1		++	+++	+++	RH, PYS: 100%	[94]
ΔGra17		++	+	+++	RH, PRU, PYS, TgC7: 100%	[95]
ΔCdpk2		++	+	+++	RH, PRU, PYS, TgC7: 100%	[99]

## Data Availability

Data sharing not applicable.
